# GCSH antisense regulation determines breast cancer cells’ viability

**DOI:** 10.1038/s41598-018-33677-4

**Published:** 2018-10-18

**Authors:** Anna Adamus, Petra Müller, Bente Nissen, Annika Kasten, Stefan Timm, Hermann Bauwe, Guido Seitz, Nadja Engel

**Affiliations:** 1 Department of Pediatric Surgery, University Hospital Marburg, Baldingerstraße, 35033 Marburg, Germany; 2 Department of Cell Biology, Rostock University Medical Center, Schillingallee 69, 18057 Rostock, Germany; 3 Department of Oral and Maxillofacial Surgery, Facial Plastic Surgery, Rostock University Medical Center, Schillingallee 35, 18057 Rostock, Germany; 4 University of Rostock, Plant Physiology Department, Albert-Einstein-Straße 3, 18059 Rostock, Germany

## Abstract

Since it is known that cancer cells exhibit a preference for increased glycine consumption, the respective glycine metabolizing enzymes are in focus of many research projects. However, no cancer associated studies are available for the Glycine Cleavage System Protein H (GCSH) to date. Our initial analysis revealed a *GCSH*-overexpression of the protein-coding transcript variant 1 (*Tv*1) in breast cancer cells and tissue. Furthermore, a shorter (391 bp) transcript variant (Tv*) was amplified with an increased expression in healthy breast cells and a decreased expression in breast cancer samples. The *Tv*1/Tv* transcript ratio is 1.0 in healthy cells on average, and between 5–10 in breast cancer cells. Thus, a GCSH-equilibrium at the transcript level is likely conceivable for optimal glycine degradation. A possible regulative role of Tv* was proven by *Tv*1-Tv*-RNA-binding and overexpression studies which consequently led to serious physiological alterations: decreased metabolic activity, release of the lactate dehydrogenase, increased extracellular acidification, and finally necrosis as a result of impaired plasma membranes. In contrast, *Tv*1-overexpression led to an additional increase in cellular vitality of the tumor cells, primarily due to the acceleration of the mitochondrial glycine decarboxylation activity. Ultimately, we provide the first evidence of a sensitive GCSH-antisense regulation which determines cancerous cell viability.

## Introduction

Glycine metabolism, particularly the glycine-to-serine conversion by the glycine decarboxylase (GLDC) and the three other proteins of the glycine cleavage system (GCS), in conjunction with serine hydroxymethyltransferase (SHMT), are functionally associated with one-carbon (C1)-metabolism for the generation of purines in nearly all organisms. With respect to cancer cell metabolism, recent studies revealed the glycine-to-serine conversion as another capability for energy generation and as a precursor for one-carbon metabolism in tumorigenic, highly proliferative cells^1–6^. Therefore, many cancer cells are not exclusively dependent on glycolysis (Warburg effect) for the supply with reducing equivalents. Rather, the glycolytic flux can be redirected from the intermediate product 3-phosphoglycerate to form serine, the precursor of glycine, cysteine and a variety of C1 compounds^1,3,4^. These novel investigations also proved that enzymes involved in the glycine-to-serine conversion are overexpressed in cancer cells to match the high demand of C1-bodies for proliferation purposes^3^. For example, the metabolic dependency on glycine is impressively illustrated by Jain *et al*.^5^, showing a preference for increased glycine consumption in 60 tumor cell lines (NCI-60 panel, CORE profiles of 219 metabolites), and Zhang *et al*.^6^ identified enzymes of the GCS as a central switching site for tumor initiating cells (TICs) in the non-small cell lung carcinoma. In this context, abnormal activation of the glycine decarboxylase correlates with a poor survival rate of lung cancer patients. In addition, glycine-dependent rapid cancer cell proliferation *via* upregulation of mitochondrial glycine biosynthesis is closely linked to the high mortality rate of breast cancer patients^3,5^. Factors such as lymph node status and tumor grade were congruent, and thus confirmed the conclusion for a poor prognosis. In summary, increased expression of mitochondrial GCS promotes the metabolism of glycine and thus tumorigenesis^1,3,5,6^. Interestingly, plant physiologists focusing on glycine metabolism in *Arabidopsis thaliana* (Arabidopsis), could proof that elevated GCSH protein (alternatively named H-protein) concentrations increase GCS activity^7^. Consequently, GCSH overexpression improved plant growth, which could likely be explained due to alleviated feedback inhibition of photosynthesis from photorespiratory intermediates^8^. However, up to now we can only hypothesize that the GCSH protein content might be involved in the proliferation behavior in plants as well as in human cells, and therefore should be studied in more detail. So far, no GCSH expression studies in correlation with tumor progression are available.

The GCS is a ubiquitously occurring multi-enzyme system consisting of four proteins: GLDC (EC 1.4.4.2, glycine decarboxylase, P-protein, 113 kDa), AMT (EC 2.1.2.10, aminomethyl transferase, T-protein, 44 kDa), DLD (EC 1.8.1.4, dihydrolipoyl dehydrogenase, L-protein, 112 kDa) and GCSH (glycine cleavage system protein H, H-protein, 19 kDa)^9^. This mitochondrial multi-enzyme system converts glycine, NAD^+^ and tetrahydrofolate (THF) to serine, NADH and *N*^5^*,N*^10^-methylene-THF, thereby releasing CO_2_ and ammonia^10,11^. The lipoamide-containing H-protein (GCSH) is an enzymatically inactive cofactor of the other three GCS enzymes and functions as oxidizing agent and shuttle of the methylamine group remaining after oxidative glycine decarboxylation from the GLDC to the AMT. Defects in the *GCSH* gene could be a cause of nonketotic hyperglycinemia in human beings^12^. The single human *GCSH* gene is located on chromosome 16 at q23.2 and composed of 5 exons spanning 13.5 kb. So far, two *GCSH* transcript variants are annotated: *Tv*1 encodes *GCSH* while *Tv*2 is a non-coding variant that lacks an alternate exon because of a frame shift^13^. Interestingly, the GCSH protein tends to dimerize and complex formation^14^. According to *in silico* data, *GCSH* expression is elevated in brain (e.g. cerebral cortex) and endocrine tissues (e.g. thyroid gland), as well as in kidney, liver, colon and sexually specific organs such as breast and prostate in human beings. In contrast, *GCSH* expression in cancer seems to be often very high in thyroid, lung, colon and breast cancer tissues (https://www.proteomicsdb.org; http://www.proteinatlas.org).

Here, we present the first expression analysis of the *GCSH* gene in human healthy and cancerous breast cells which directed us to the identification of a previously not described regulatory mechanism. This is an antisense mechanism that regulates cellular GCSH dimerization and possibly the flux via the glycine cleavage system by a newly identified transcript variant, namely Tv*. This evaluation could be of major interest since breast cancer has the highest incidence rate of all cancers in women worldwide^15^. Treatment strategies depend on the histological subtype and receptor expression status. Positive testing for estrogen, progesterone, and the human epidermal growth factor receptor-2 allows effective therapy with Tamoxifen, Anastrozole, or Trastuzumab^16,17^. No detectable expression of these three receptors is associated with poor prognosis and difficult treatment options. To include both, receptor-positive and -negative breast cancer cells, we used in this study two triple negative breast cancer cell lines (BT-20, MDA-MB-231) and one luminal receptor positive cell line MCF-7. MCF-10A and MCF-12A were used as healthy control breast cells^18^.

## Results

### GCSH protein is overexpressed in both, breast cancer tissue and breast cancer cell lines

**Figure 1:**
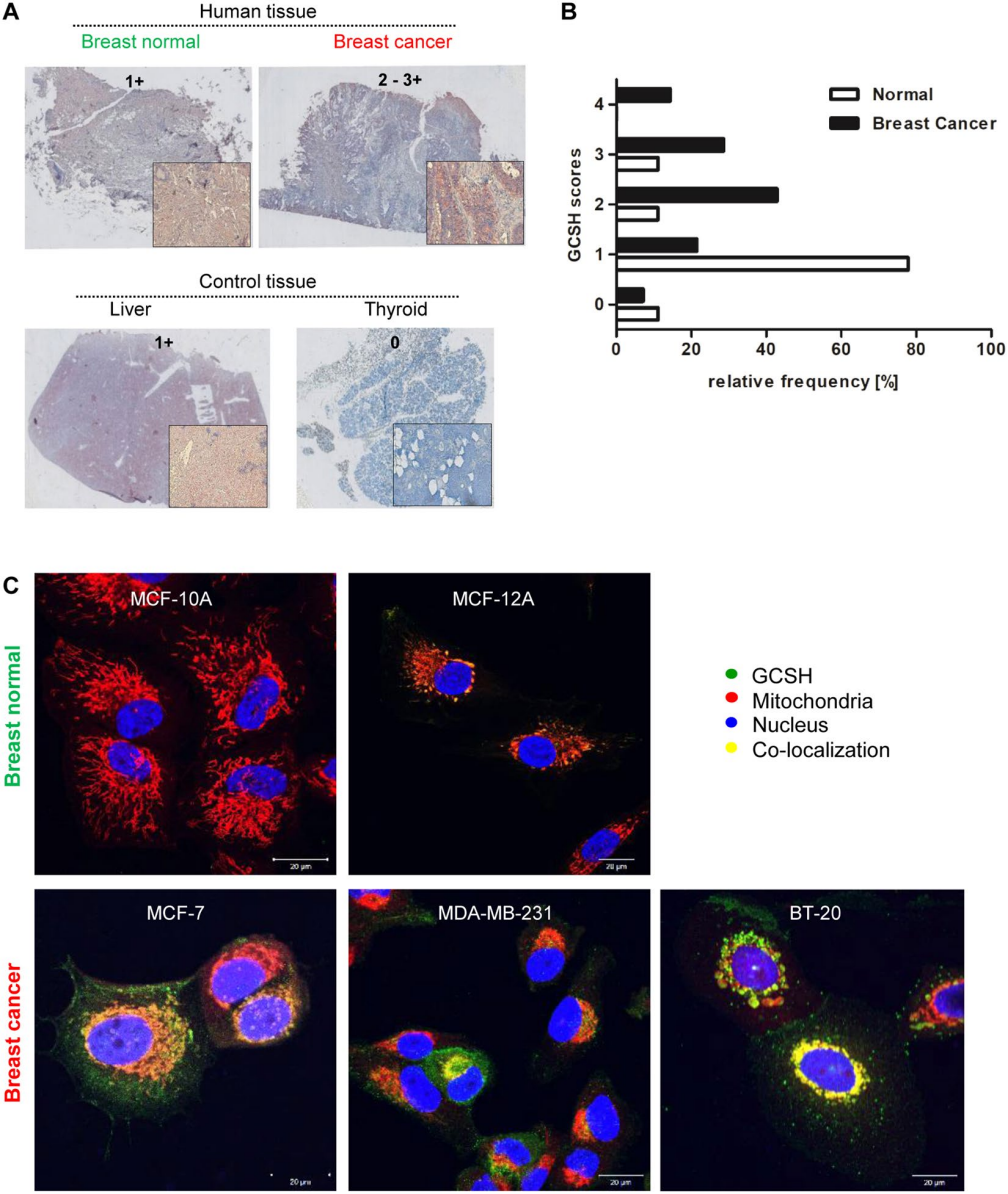
GCSH expression analysis. (**A**) GCSH expression *in vivo*. Representative images of GCSH immunohistochemistry staining in normal and breast cancer tissue samples in comparison with high (liver) or low (thyroid) GCSH-expressing organs. Staining intensity grade is scored from 0 to 4+. Higher magnifications are shown in boxes in the right upper corner of every image. (**B**) Relative frequency of GCSH scores of IHC stained normal and breast cancer tissues. Higher GCSH scores and thus also higher GCSH expression occurs significantly more frequently in breast cancer. Normal breast tissue n = 9. Breast cancer tissue n = 14. (**C**) GCSH expression *in vitro*. GCSH immunofluorescence staining of two non-tumorigenic, epithelial breast cell lines (MCF-10A, MCF-12A) in comparison with three established breast cancer cell lines (MCF-7, MDA-MB-231, BT-20). A co-localization of GCSH and mitochondria was found. Notably, breast cancer tissue and all cancer cell lines revealed a strong GCSH overexpression compared to normal breast tissue and non-tumorigenic cell lines.

To evaluate GCSH expression *in vivo* as well as *in vitro*, paraffin-embedded normal breast and breast cancer tissue (Fig. 1A,B) as well as three commercially available breast cancer cell lines (MCF-7, MDA-MB-231 and BT-20; Fig. 1C) in comparison with non-tumorigenic controls were immunolabeled with a monoclonal, full length GCSH-specific antibody. In general, non-tumorigenic, resting mammary tissue revealed a low GCSH expression. Only in metabolic active regions e.g. ducts, small amounts of GCSH protein (score 1+) could be verified. In contrast, the GCSH protein could be detected equally distributed in the whole mammary carcinoma tissue, with an expression score ranging from 2+ to 3+. In all samples, the GCSH protein is associated with mitochondria as visualized by a hematoxylin-eosin counterstaining. Some of the detected GCSH protein in the non-tumorigenic tissue appear to be also associated with the nucleus (Personal communication Prof. Erbersdobler, Department of Pathology, University of Rostock, Rostock, Germany). Similar results were obtained by GCSH-immunofluorescence labeling of breast cancer cell lines (Fig. 1C). A GCSH-overexpression could be confirmed for the breast cancer cell lines MCF-7 and BT-20. Interestingly, MDA-MB-231 showed low GCSH-expression, comparable with that of non-tumorigenic control cell lines (MCF-12A and MCF-10A). Association between GCSH and mitochondria was confirmed by co-localization studies, too.

**Figure 2:**
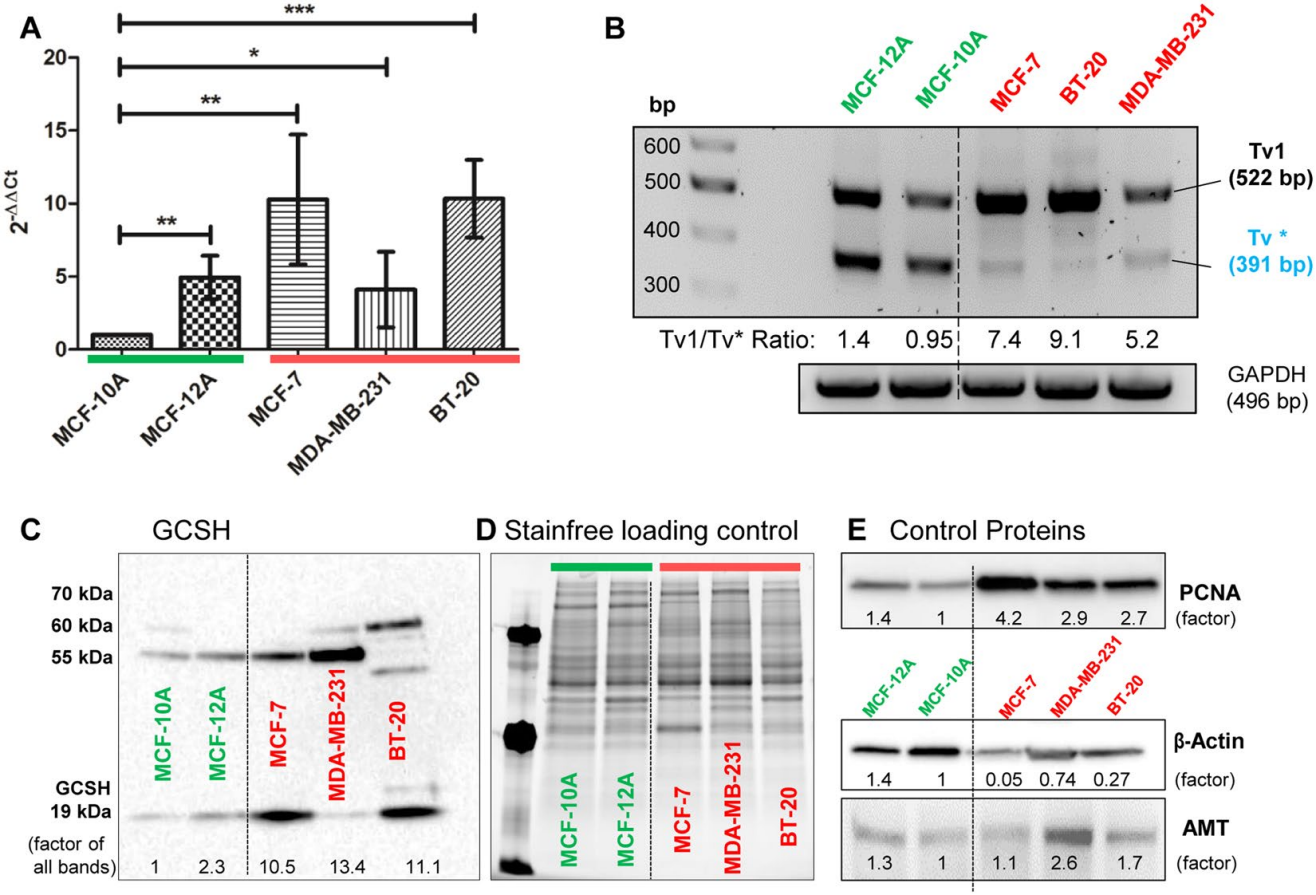
GCSH transcript and protein analysis. (**A**) qRT-PCR analysis using GCSH *Tv*1 specific primers showed an increase in GCSH *Tv*1 expression in all three breast cancer cell lines (red) as well as in the non-tumorigenic control cell line MCF-12A compared to the normal breast cells of MCF-10A (green). MCF-7 and BT-20 revealed the highest GCSH *Tv*1 transcript levels. Mean ± SD, n = 5, ***P < 0.001, **P < 0.01, *P < 0.05, significantly different compared to MCF-10A, unpaired *t*-test. (**B**) RT-PCR analysis using GCSH *Tv*1 full length primers confirmed the qRT-PCR results. A 2^nd^ transcript variant (Tv*) with 391 bp in size was amplified and significantly lower expressed in all cancer cell lines. *Tv*1/Tv* ratios ranged from 5–10 for the cancer cells. (**C**) GCSH protein content in all 5 cell lines was visualized by western blotting. Native GCSH protein (19 kDa) content was increased for MCF-7 and BT-20, as analogous to the transcript analysis. However, further signals could be detected at ~58 kDa and ~65 kDa. The densitometrically calculated sum factor revealed an approximately 10-fold higher GCSH protein content in all breast cancer cell lines. (**D**) Stainfree imaging to guarantee identical loaded protein contents, used for the protein content normalization additionally. (**E**) Control Western blots of PCNA (proliferating nuclear antigen), ß-actin (intended as housekeeping protein) and AMT (aminomethyltransferase, direct GCSH interacting enzyme). Protein contents were calculated densitometrically and set in relation to MCF-10A protein levels.

These expression results were verified by qRT-PCR and semi quantitative RT-PCR (Fig. 2A,B), as well as immunoblotting (Fig. 2C). Indeed, increased *GCSH* expression levels of the breast cancer cell lines MCF-7 and BT-20 were also calculated on transcript and protein levels, with MDA-MB-231 as an exception. A higher GCSH protein content could also be verified by immunoblotting, but on a higher molecular weight level (~60 kDa). As internal controls PCNA (proliferating nuclear antigen), ß-actin (intended as housekeeping protein), and AMT (aminomethyltransferase: a direct GCSH interacting protein, also names T-protein) were used (Fig. 2E). All three breast cancer cell lines displayed approximately 10-fold higher GCSH protein expression as well as significantly boosted PCNA contents and moderate lowered ß-actin levels. Again, MDA-MB-231 exceptionally showed a higher AMT-protein content. However, identical loading of soluble proteins was guaranteed by stain-free technology (Fig. 2D). Protein expression factors were determined densitometrically and normalized to the non-tumorigenic control cell line MCF-10A, which was arbitrarily set to 1. Given the existence of two *GCSH* transcript variants exist, primers flanking the full-length *GCSH* transcript were used to confirm qRT-PCR results by reverse transcriptase PCR (Fig. 2B). All cell lines expressed the full-length transcript variant *Tv*1 (522 bp) to a comparable extent as detected by qRT-PCR. Additionally, a second, shorter variant could be amplified (~350 bp), further designated as Tv*. Unexpectedly, Tv* was significantly downregulated in all cancer cell lines.

### Transcript variants *Tv*1 and Tv* display an antisense regulation mechanism

**Figure 3:**
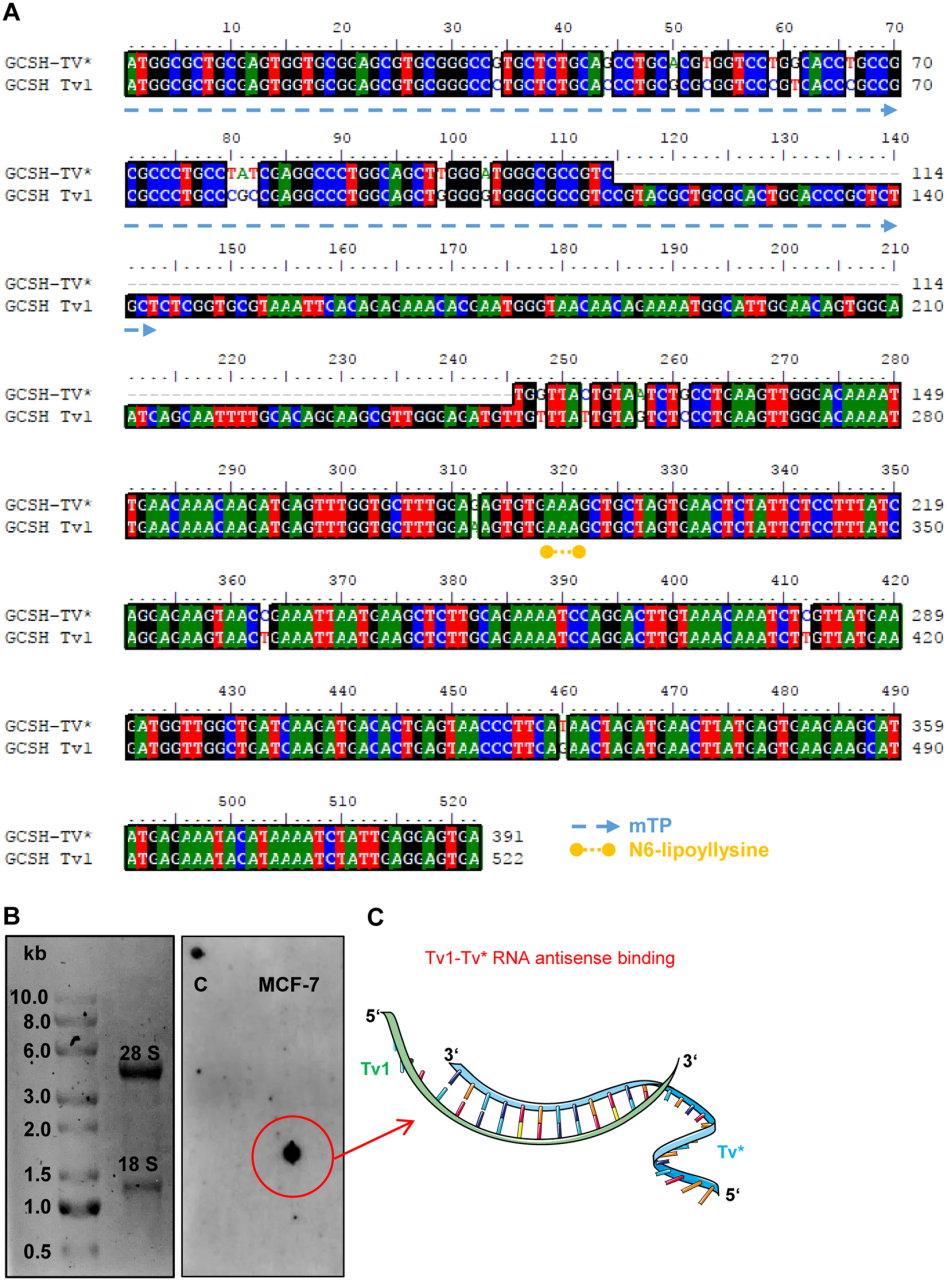
Sequence alignment and RNA-binding study. (**A**) Nucleotide sequence alignment between the GCSH transcript variant 1 (*Tv*1, coding for the GCSH protein), the newly identified transcript variant (Tv*). *Tv*1 to Tv* is 97% identical. (**B**) Northern blotting demonstrated a positive antisense binding of the Tv* to isolated RNA of the cancer cell line MCF-7. (**C)** Model of *Tv*1-Tv* RNA antisense binding. Original figure ‘Transcrpition’ by Servier Medical Art (https://smart.servier.com/smart_image/transcription/) is licensed under a Creative Commons Attribution 3.0 Unported License. This modified figure is licensed under CC BY by Nadja Engel.

DNA sequencing and the usage of basic local alignment search tools (BLAST) annotated the transcript variant Tv* as the *homo sapiens* GCS H-protein (aminomethyl carrier) pseudogene 3 (GCSHP3), non-coding RNA (NCBI accession no.: NR_033248.1) with 100% nucleotide sequence identity to *Tv*1. Tv* sequencing revealed a transcript length of 391 bp (Fig. 3A). Nucleotide identity between Tv* and *Tv*1 was calculated to 97%. Obviously, Tv* lacks 30 bp of the N-terminal mitochondrial transit peptide region which makes mitochondrial localization unlikely. However, based on the altered transcriptional expression between non-tumorigenic and cancerous cells, a regulative function for Tv* would be possible. Speculating on an antisense regulating mechanism, we analyzed whether *Tv*1 and Tv* RNA sequences can bind each other. Using northern blotting analysis, a stable *Tv*1-Tv*-RNA-binding was observed, and, moreover, revealed that Tv* is present in antisense orientation in the breast cancer cell line MCF-7 (Fig. 3B).

### Transient Tv* elevation reduces *Tv*1 transcript and protein content

**Figure 4:**
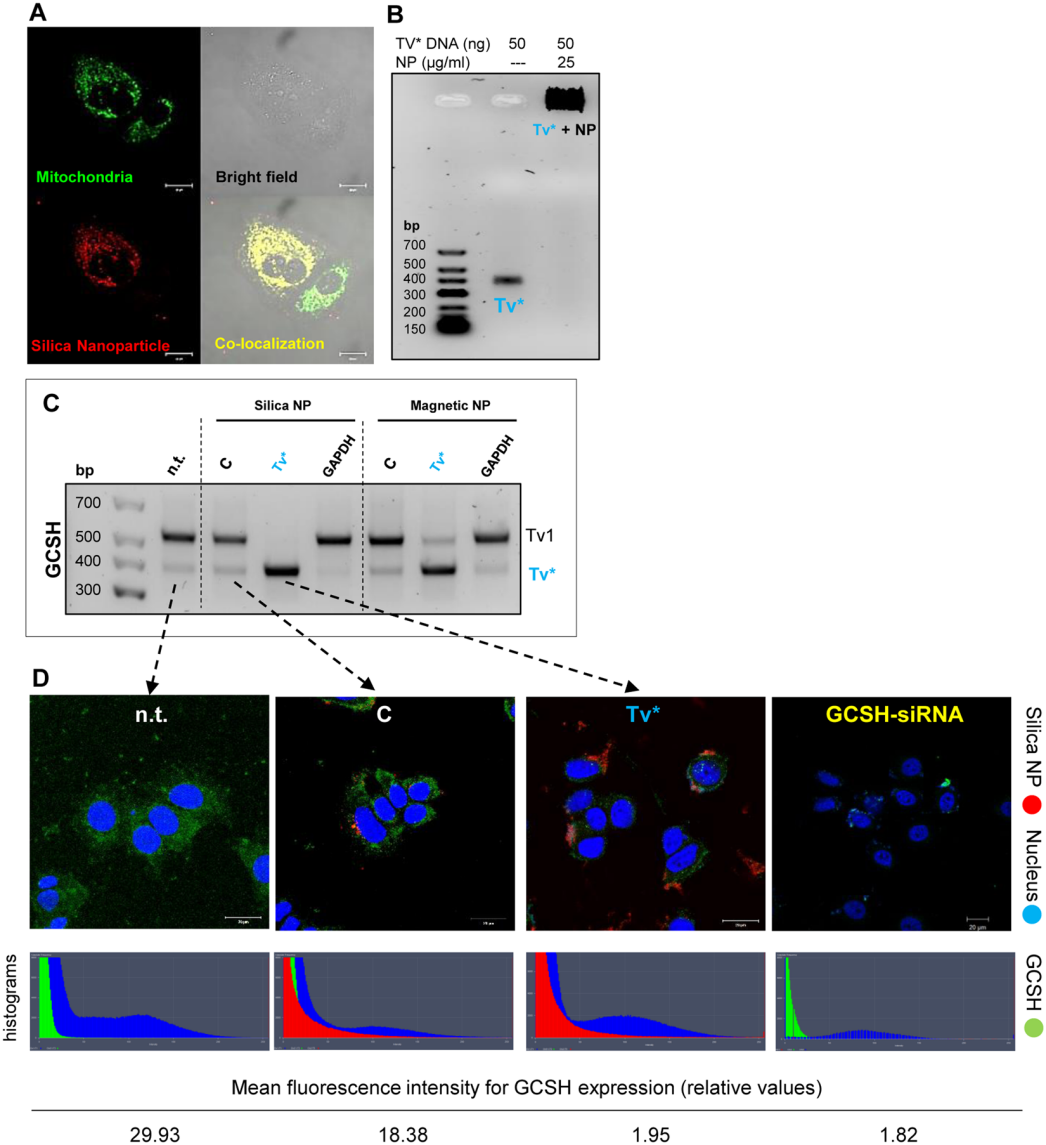
Nanoparticle-mediated transient Tv*-overexpression. (**A**) Co-localization (yellow) between mitochondria (green) and nanoparticles (red) was found, indicating a direct association of the nanoparticles with mitochondria. (**B**) Evidence of the stable binding between Tv* and nanoparticles. All of the 50 ng Tv*-DNA was completely bound to the nanoparticles which cannot pass the agarose gel. (**C**) RT-PCR of GCSH transcripts *Tv*1 and Tv* of non-transfected (n.t.), mock-transfected (**C**), Tv*-transfected or GAPDH-transfected MCF-7 cells. Results were similar with silica and magnetic nanoparticles: Tv*-overexpression decreased *Tv*1 expression, and Tv* was found as the most prominent band. In general, control transfection (nanoparticles, w/o DNA) did not alter the GCSH transcript profile. (**D**) Immunofluorescent visualization and fluorescence intensity histograms of the endogenous GCSH protein content using a monoclonal GCSH antibody (green), fluorescence of the nanoparticles (red) and cell nuclei (blue) in non-, control- and Tv*-transfected MCF-7 cells. Tv*-overexpressed cells and GCSH-siRNA knockdown cells. Notably, GCSH fluorescence intensity was obviously reduced in Tv* transfected cells and GCSH-siRNA knockdown cells.

The assumed antisense regulation mechanism was analyzed by nanoparticle driven Tv*-overexpression in all three cancer cell lines and in both non-tumorigenic control cell lines (see SFig. 1). All further experiments were conducted with the breast cancer cell line MCF-7, presenting the most common breast cancer subtype (positive for estrogen and progesterone receptor expression). For transient Tv*-transfection purposes, silica and magnetic, red fluorescent nanoparticles were chosen. These nanoparticles mediated mitochondrial localization after cellular internalization, visualized by yellowish co-localization with green mitochondrial tracker (Fig. 4A). Stable binding of Tv*-DNA with nanoparticles was verified by DNA-agarose electrophoresis (Fig. 4B), and therefore its suitability for transfection is guaranteed. Hence, breast cancer cells were next transfected with GCSH-Tv*-loaded nanoparticles. A considerable increase of Tv* PCR products was detected by RT-PCR, with a simultan decrease in the amount of *Tv*1 PCR products (Fig. 4C). This result represents the first indication for an antisense-regulative mechanism. In parallel, GCSH protein expression of Tv*-transfected cells was analyzed by confocal laser-scanning microscopy (Fig. 4D). Mock transfected cells revealed a mitochondria-associated localization of red fluorescent nanoparticles. Also, GCSH protein distribution in mock transfected cells was not altered in comparison to the non-transfected MCF-7 cells. In contrast, Tv*-transfected MCF-7 cells showed an accumulation of red nanoparticles around the nucleus and a lowered GCSH protein content in general.

### Stable transfection with Tv* decreases cell viability

**Figure 5:**
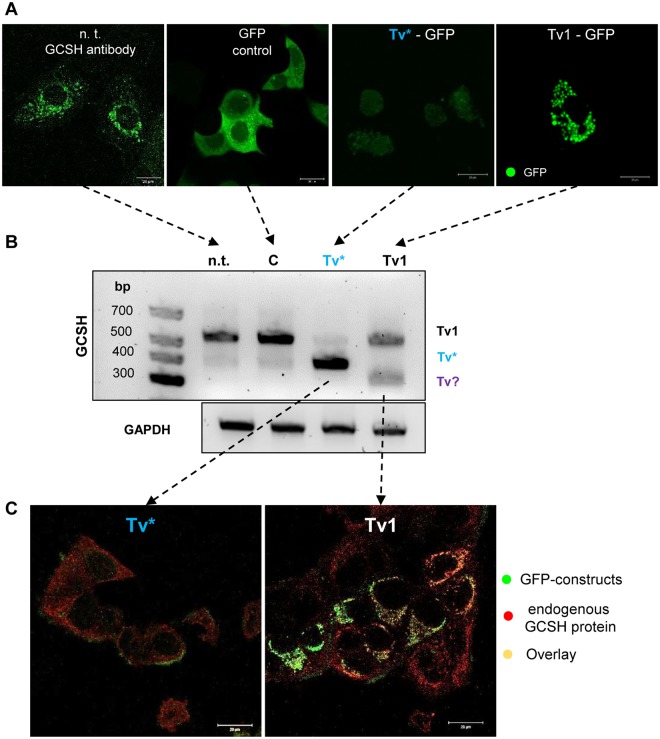
Stable overexpression of *Tv*1 and Tv*. (**A**) Plasmid mediated overexpression of *Tv*1 and Tv* in comparison with the GCSH-labeled, non-transfected (n.t) or GFP-transfected (C) MCF-7 cells. Notably, Tv*-overexpression mediated a weak, cytosolic expression whereas *Tv*1-overexpressors displayed a strong, mitochondrial GFP-signal. Those mitochondria are much bigger and located around the cell nucleus. (**B**) RT-PCR of GCSH transcripts *Tv*1 and Tv* of non- (n.t.), control- (C), Tv*- and *Tv*1-transfected MCF-7 cells. Tv*-overexpression revealed comparable results to nanoparticle mediated transfection: low *Tv*1 and high Tv* signals. Surprisingly, *Tv*1-overexpression did not increase the *Tv*1 content. Furthermore, no Tv* amplificat could be detected but instead a third ~300 bp transcript variant (*Tv*?) appeared. (**C**) Simultaneous visualization of *Tv*1- or Tv*-GFP-expression (green) and endogenous GCSH content (red) by confocal laser-scanning microscopy.

Nanoparticle mediated Tv*-overexpression was used as a transient, primary test system, while physiological alterations can only be monitored with stably transfected cells. Thus, the *GCSH* transcript variant *Tv*1 as well as Tv* were cloned in frame with the GFP-ORF of the vector pCMV6-AC-GFP (SFig. 2) and transfected into the cells (Fig. 5A). As positive control, the GFP-vector was used, known to cause cytoplasmic green fluorescence in MCF-7 cells. The Tv*-GFP-construct revealed a minor cytoplasmic fluorescence, which indicates the absence of the full-length mitochondrial transit peptide. However, *Tv*1-GFP overexpressing cells showed the expected mitochondrial distribution (Fig. 5A, right). Notably, mitochondria showed a round, swollen shape in comparison with non-treated MCF-7 cells (Fig. 5A, left). Tv*-overexpression modulated the *Tv*1/Tv* ratio to a similar extent as transient particle-driven Tv*-expression, that reduced *Tv*1 and increased Tv* transcript (Fig. 5B, compare with Fig. 4C). In contrast, overexpression of *Tv*1-GFP did not significantly increase the *Tv*1 transcript content but induced formation of a third transcript variant (~300 bp). Simultaneous visualization of the endogenous GCSH protein (red) and the GCSH-GFP-constructs (green) confirmed low cytosolic expression of Tv* and high mitochondrial expression of *Tv*1 (Fig. 5C), too.

**Figure 6:**
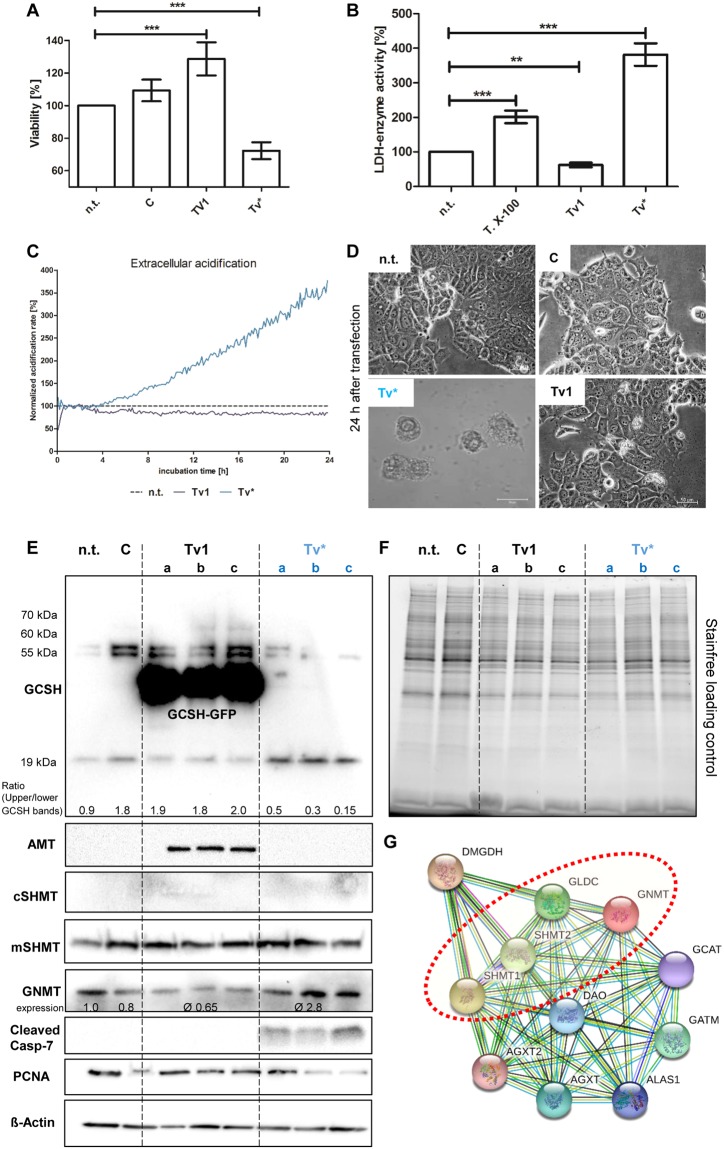
Physiological alterations of *Tv*1- and Tv*-overexpressors. (**A**) Metabolic cell viability was significantly increased in *Tv*1- and significantly reduced in Tv*-overexpressors in comparison to the non- and control-transfected cells; n = 8. (**B**) Cell membrane impairment was measured by the release of the cytosolic lactate dehydrogenase (LDH). The non-transfected control was set to 100%. Triton X-100 (T.X-100) incubation functioned as positive control. *Tv*1-overexpressors showed a slight decrease and Tv*-overexpressors a 4fold increase in extracellular LDH activity; n = 8. (**C**) The extracellular acidification was measured by real-time online monitoring of living MCF-7 cells over 24 h. Also, a 4fold increase was detected. (**D**) Brightfield images of non- (n.t.), control- (C), Tv*- and *Tv*1-transfected MCF-7 cells. Only Tv*-overexpressors showed a necrotic phenotype. (**E**) Immuno blots of GCSH, AMT, CSHMT, mSHMT, GNMT, and cleaved caspase-7, PCNA, ß-actin proteins in non-treated (n.t.), mock-treated (C), three individually transfected *Tv*1- and Tv*-overexpressor MCF-7 cell populations. (**F**) Stain-free image functioning as loading control. (**G**) Network of protein interactions created with, https://string-db.org shows evidence and data-based co-expression (black line) of GLDC, GNMT, cSHMT and mSHMT (framed in red). All data and download files in STRING are freely available under a ‘Creative Commons BY 4.0’ license.

The physiological impact of *Tv*1 and Tv*-overexpression was examined in GFP-sorted MCF-7 cells. First, the impact on the general cell viability was checked (Fig. 6A). Whilst overexpression of *Tv*1 induced a significant increase (+30%), overexpression of Tv* mediated a decrease (−40%) in the metabolic viability in comparison with corresponding control treatments. This observation was verified by lactate dehydrogenase (LDH) release measurements, accounting for a first indication of plasma membrane permeabilization, a measure of necrosis or apoptosis induction (Fig. 6B). *Tv*1-transfected cells harbored a significantly reduced extracellular LDH activity while Tv* showed an upregulation of up to 400%. Metabolic real-time monitoring of viable MCF-7 breast cancer cells confirmed the rise of the extracellular acidification over time (Fig. 6C). 24–30 h after Tv*-transfection, tumor cells are no longer viable. In general, membrane impairment leads to leakage of intracellular molecules and enzymes like the abundant LDH. MCF-7 cells first lost their normal shape and subsequently died by damaged plasma membrane (Fig. 6D). By immunoblotting it was shown that Tv* reduces the multimerized GCSH bands and increases the monomeric GCSH band at 19 kDa. This result clearly proves that Tv* influences more the GCSH expression mainly by impairing multimerization events. Next, a conclusion can be drawn between the GCSH expression ratio of the multimerized upper bands and the monomeric 19 kDa bands. The lower ratio between those bands is the higher is the impact on apoptosis induction (cleaved caspase 7) and proliferation inhibition (PCNA-proliferating nuclear antigen). Further, immunoblotting showed that *Tv*1 overexpression, here fused with GFP-protein, also enhanced AMT and lowered the glycine N-methyltransferase protein expression (Fig. 6E). Expression of the mitochondrial serine hydroxymethyltransferase (mSHMT) was not altered at any cells. The cytosolic SHMT (cSHMT) was not expressed although co-expression was predicted for GLDC, GNMT and both SHMT variants (Fig. 6F). In contrast, Tv* overexpression induced caspase-7 cleavage and a slight reduction of PCNA (Fig. 6F). Loading control was achieved by stain free imaging prior and after blotting as well as detection of ß-actin. Finally, the impact on GCS activity was examined by determination of the glycine-serine ratio (Table 1). Notably, reduction of the GCSH content, achieved either by the transfection of Tv* or siRNAs, resulted in an approximately 20% lowered glycine levels. On the contrary, overexpression of *GCSH* variant *Tv*1 elevated the glycine levels up to 25%. The glycine-to-serine ratio revealed a shift in glycine-to-serine equilibrium after Tv* transfection, since the ratio between both amino acids decreased from 2.4 to 1.7 on average.

**Table 1:** Cellular glycine (Gly) and glycine/serine (Gly/Ser) ratios of MCF-7 cells 24 h after transfection with Tv1, Tv* plasmids or GCSH-siRNAs measured by LC-MS. Gly control was set to 1.

	C	*Tv*1	Tv*	*GCSH*-siRNA
Gly	1.00 ± 0.13	1.27 ± 0.17	0.83 ± 0.21	0.81 ± 0.23
Gly/Ser ratio	2.43 ± 0.16	2.23 ± 0.19	1.74 ± 0.20*	2.11 ± 0.23

Mean ± SD, n = 7–10, ^*^P < 0.5, significantly different compared to control (C), unpaired t-test.

## Discussion

Given the fact that cancer cells are characterized by a high demand for C1 bodies to ensure continuous growth, enzymes of glycine metabolism, such as GLDC, are overexpressed in tumorigenic tissue^6,19^. In addition, a combination of low-GLDC/negative-HIF-1α expression has emerged as early prognostic factor for long-term survival in non-small lung cell cancer^20^. However, GLDC is only one out of four protein components of the GCS, which is ubiquitously essential for one-carbon metabolism^9^. Another protein of this system is the GCS H-protein (GCSH) which forms the core of the entire GCS, since it functionally connects all GCS enzymes via its lipoyl arm^9^. Under *in vitro* conditions, GCS activity is stimulated by external GCSH supply. Consequently, a ~50-fold stimulation of the glycine–bicarbonate exchange rate, relative to the rate measured in the absence of exogenous H-protein, could be reached^7^. *In vivo* this phenomenon was also verified in the model plant Arabidopsis. Transgenic GCSH overexpressors showed accelerated glycine turnover, yielding higher biomass production^8^. This fundamental finding raises the question, if higher GCSH protein amounts have an impact on the proliferation of breast cancer cells?

Our first result confirmed that the GCSH protein is overexpressed in breast cancer tissue and breast cancer cells. Unexpectedly this overexpression does not depend on the histological subtype. Both, luminal, hormone receptor positive, and, basal, triple negative breast cancer cells exhibited a 10-fold increased GCSH content (Fig. 2C), which is congruent with the reported higher GCS in various cancer types^6^. However, we here demonstrate for the first time that additional GCSH, which very likely results in higher overall GCS activity, strengthens the viability of the breast cancer cells and therefore forces tumorigenesis (Fig. 6). This finding is further supported by the fact that higher GSCH contents correlate with a poorer long-term and relapse free survival rate (SFig. 3).

Secondly, due to the identification of a second transcript variant (Tv*), which is downregulated in all tested breast cancer cell lines, we hypothesized that Tv* could be important for the regulation of the cellular GCSH content, perhaps via antisense repression (Fig. 2). This hypothesis was verified by RNA-binding studies and Tv*-overexpression. Antisense binding of Tv* to *Tv*1 was shown by northern blotting (Fig. 3). Physiologically, transfection with Tv* mediated a weak cytosolic expression that causes reduced cell viability and cell membrane impairment of MCF-7 cells. Excess of Tv* reduces the expression of *Tv*1 and induces necrosis of the breast cancer cells within 24–30 h, which was verified by caspase-7 cleavage, an executioner protein of apoptosis and necrosis (Fig. 6). This result implies that the *Tv*1/Tv* ratio decisively determines *GCSH* expression and subsequently the capacity for providing C1-units to several biosynthetic pathways. Furthermore, measurement of the cellular glycine and serine levels implicate that the GCSH content significantly affects the cellular glycine-serine equilibrium (Table 1). To our surprise Tv* overexpression did not significantly change glycine levels, and the small change is in the opposite direction as expected - one expects increased glycine levels due to decreased glycine degradation. On explanation is that the amount and/or activity of the glycine producing serine hydroxymethyltransferase (SHMT) is increased. The cytoplasmic and mitochondrial SHMTs catalyze the conversion of serine to glycine with the transfer of β-carbon from serine to tetrahydrofolate (THF) to form 5, 10-methylene-THF. SHMTs are directly interacting with proteins of the glycine decarboxylating system and thus with GCSH, too (Fig. 4G). By western blotting we showed that the amount of the mitochondrial SHMT (mSHMT) was not altered and the cytosolic SHMT protein was not expressed. Therefore, we checked several glycine degrading and converting enzymes. On protein level, we found that the glycine N-methyltransferase (GNMT) expression is repressed in *Tv*1 overexpressors. Thus, one could speculate that glycine is not further converted into N-methylglycine in *Tv*1 overexpressors and therefore accumulates intracellularly. Tv* cells overexpress GNMT protein, as a result, more glycine is metabolized and thus measured in reduced concentrations by LC-MS. However, the effects of such a shift in the Gly-Ser balance on cell vitality and its consequences must be determined in the future studies.

Nevertheless, Jain *et al*.^5^ proved that glycine metabolism plays an essential role for high cell proliferation rates. In addition, metabolic studies showed that fast-growing cells take up more glycine. On the other hand, non-proliferative active cells even released glycine into the medium. Radiolabeling of the ingested glycine has been shown to be used directly for purine synthesis. And here we are, glycine degradation by the glycine decarboxylation system is the primary C1 unit donor for purine synthesis.

Collectively, our results suggest that the cellular GCSH content is a key factor that determines the metabolic state and viability of cells, including tumorigenesis, and thus is a potent tumor marker for highly proliferative breast cancers. The cellular GCSH content itself is sensitively regulated by antisense binding of a 391 bp *GCSH* transcript variant Tv* to the coding transcript variant *Tv*1. We hypothesize that overexpressed Tv* directly binds to the coding transcript variant *Tv*1 whereby GCSH translation is restrained. No GCSH protein could be imported into the mitochondria whereby overall mitochondrial GCS activity is lowered over time leading to plasma membrane impairment and necrosis. Thus, it is likely to argue that the GCSH content could be a key player in tumorigenesis, and, therefore, a potent metabolic tumor marker.

## Material and Methods

### Cell lines and culture conditions

Experiments were performed as reported in our previous work^21^. Briefly, the non-tumorigenic mammary epithelial control cell lines MCF-10A (ATCC^®^ CRL-10317^™^) and MCF-12A (ATCC^®^ CRL-10782^™^) were grown in Dulbecco’s modified Eagle’s medium Ham’s F12 without phenol red (Invitrogen, Darmstadt, Germany) containing 10% horse serum (PAA Laboratories GmbH, Munich, Germany), the Mammary Epithelial Cell Growth Medium Supplement Pack (Promo Cell, Heidelberg, Germany) including 0.004 ml/ml bovine pituitary extract, 10 ng/ml epidermal growth factor (recombinant human), 5 mg/ml insulin (recombinant human), 0.5 mg/ml hydrocortisone and 1% gentamycin (Ratiopharm GmbH, Ulm, Germany). The breast cancer cell lines MCF-7 (ATCC^®^ HTB-22^™^; estrogen and progesterone receptor positive), BT-20 (ATCC^®^ HTB-19^™^) and MDA-MB-231 (ATCC^®^ HTB-26^™^), both triple negative cancer cell lines were cultured in Dulbecco’s modified Eagle’s medium (Gibco, Paisly, UK) with 10% fetal bovine serum (PAN Biotech GmbH, Aidenbach, Germany) and 1% gentamicin (Ratiopharm GmbH, Ulm, Germany). Confluent cells were treated with 0.05% trypsin – 0.02% EDTA. The medium was changed every 2 days. Cell line authentication was performed by Seqlab Sequencing Laboratories (Göttingen, Germany).

### Expression analysis

Total RNA was extracted with NucleoSpin® RNA II (Machery-Nagel, Düren, Germany) and cDNA was produced with the First Strand cDNA Synthesis Kit (Thermo Fisher Scientific Inc., Rockford, IL, USA). GCSH full length coding sequence was amplified with primer pair fw: 5′-ATGGCGCTGCGAGTGG-3’ and rv: 5′-TCACTCCTCAATAGATTTTATG-3′ using Dream Taq^TM^ Green PCR Master Mix (Thermo Fisher Scientific Inc., Vilnius, Lithuania). qRT-PCR was performed with primer pair fw: 5′-GTCTCCCTGAAGTTGGGACA-3′ and rv: 5′-TCTGAAGGGTTACTCAGTGTCA-3′ using iTaq^TM^ SYBR^®^ Green Supermix (Bio-Rad Laboratories Inc., Hercules, USA) in the iQ^TM^5 Multicolor Real-Time PCR Detection System (Bio-Rad, München, Germany). Relative gene expression was normalized to the GAPDH housekeeping gene: fw: 5′-CAAGGTCATCCATGACAACTTTG-3′ and rv: 5′-GTCCACCACCCTGTTGCTGTAG-3′. Sequencing of both transcript variants was done by Seqlab Sequencing Laboratories (Göttingen, Germany).

### Protein expression analysis

Western blotting procedure was performed as already described^22^. For protein detection, primary antibodies anti-GSCH (#16726-1-AP; Proteintech Europe, Manchester, UK), anti-GAPDH (#D16H11; Cell Signaling, USA), anit-PCNA (#sc-56; Santa Cruz, USA); anti-β-Actin (#4970; Cell Signaling, USA), anti-AMT (#sc-99267; Santa Cruz, USA), anti-cSHMT (#sc-365203; Santa Cruz, USA), anti-mSHMT (#sc-390641; Santa Cruz, USA), anti-GNMT (#sc-166834; Santa Cruz, USA) were incubated overnight at 4 °C followed by labeling with a horseradish peroxidase (HPR)-conjugated secondary antibody (Dako, Glostrup, Denmark) for 1 h at room temperature. Band intensity was analyzed densitometrically with the Molecular Imager ChemiDoc XRS and Image Lab 3.0.1 software (Bio-Rad, München, Germany).

### Immunofluorescence and immunohistochemistry (IHC)

Experiments were performed as reported in our previous work^21^. Briefly, the cells were grown on Menzel-Gläser coverslips (Thermo Fisher Scientific Inc., Schwerte, Germany) or in Ibidi dishes/ slides (Ibidi GmbH, Martinsried, Germany), fixed in 4% paraformaldehyde (Santa Cruz, Dallas, USA), permeabilized with 0.1% Triton X-100 (Santa Cruz, Dallas, USA) and labeled with anti-GCSH primary antibody (#16726-1-AP; Proteintech Europe, Manchester, UK) and Alexa Fluor 488 dye/Alexa Fluor 594 dye secondary antibody (Thermo Fisher Scientific Inc., USA). Mitochondria were labeled with MitoTracker® RedCMXRos or Green FM (Life Technologies Europe BV, Bleiswijk, Netherlands), counter-stained with Hoechst (PanReacAppliChem, Darmstadt, Germany) and investigated with a confocal laser-scanning microscope (LSM780, Carl Zeiss, Jena Germany). Notably, images were taken at identical device settings to guarantee comparable results. The expression intensity and distribution of GCSH protein was determined in healthy (GTX24324, GeneTex, Irvine, USA) and tumorigenic (GTX24701, GeneTex, Irvine, USA) human breast tissue. Murine liver and human thyroid tissue served as controls. Tissues were formalin-fixed, paraffin-embedded, cut, labeled with a GCSH primary antibody (#16726-1-AP; Proteintech Europe, Manchester, UK) in a 2% BSA solution followed by a secondary antibody−labeled, polymer−horseradish peroxidase (HRP) (Dako Envision+ Kit; Dako, Glostrup, Denmark). AEC (3-amino-9-ethylcarbazol) served as chromogenic agent. All sections were counterstained with Mayer’s hemalum solution (Merck KGaA, Darmstadt, Germany). Each section was digitalized by using an AxioImager.M2 equipped with an integrated XY-scanning device and a MRc camera (all from Carl Zeiss Microscopy GmbH, Göttingen, Germany). Statistical evaluation of *in vivo* GCSH protein expression was performed by semi quantitative scoring (0 = no, 1+ = low, 2+ = medium, 3+ = strong and 4 + = very strong GCSH expression).

### *GCSH* overexpression plasmids and *GCSH*-siRNAs

A *GCSH*-cDNA-GFP-tagged clone (transcript variant 1; NCBI accession no: NM_004483) was purchased from OriGene (#RG217886, Rocville, USA). Transcript variant Tv* of MCF-12A was cloned in frame into the same vector by using the restriction sites SgfI and MluI. GCSH full length coding sequence for the GCSH cloning vector was amplified with primer pair fw: 5′-AAGCGATCGCCATGGCGCTGCGAGTG-3′ and rv: 5-AAACGCGTCTCCTCAATAGATTTTATG-3′ using Universe Hot Start High-Fidelity DNA polymerase Mastermix (Biotool, Munich, Germany) and MyCycler^TM^ thermal cycler (Bio-Rad, München, Germany) to visualize full length of GCSH *Tv*1 and GCSH Tv*. The GCSH-Tv* fragment and GCSH-*Tv*1-pCMV6-AC-GFP vector were cut on SgfI and MluI restriction sites (Thermo Fisher Scientific Inc., Waltham, USA). Positive transformed DH5α *E.coli* cells were selected by restriction and sequencing (Seqlab Sequencing Laboratories, Göttingen, Germany). 3 × 10^6^ cells of breast cancer cell lines MCF7, BT20, MDA-MB-231 as well as control cell lines MCF10-A and MCF12-A were transfected with 1 μg/ml GCSH (*Tv*1 or Tv*) plasmid DNA using the CLB-Transfection System (Amaxa Nucleofection 6097129; Lonza, Cologne, Germany). As positive control the Pmax GFP^TM^ vector (Lonza, Cologne, Germany) was used. Transfection efficiency was controlled with a confocal laser-scanning microscope. To generate stable transfected cells, GFP negative cells were eliminated by fluorescence-based cell sorting (BD FACSAria, Heidelberg, Germany). 3 unique 27mer GCSH-siRNAs (GCSH Human siRNA Oligo Duplex (Locus ID 2653) were purchased from Origene (#SR301770, Rocville, USA) and were used according the manufacture’s protocol.

### Nanoparticle mediated transfection

Sicastar®-redF and BNF-Starch-redF (micromod Partikeltechnologie GmbH, Rostock, Germany) are amorphous silica nanoparticles and magnetic bionized nanoferrite nanoparticles, respectively, both 100 nm in size, precoated with amino groups (NH_2_), and labeled with a red fluorophore (Sicastar: ex. λ = 569 nm, BNF: ex. λ = 552 nm). The cell-internalization of magnetic BNF nanoparticles was stimulated by a conventional permanent magnet. To prevent nanoparticle aggregation, all nanoparticle associated experiments were performed in serum-free medium Dulbecco’s modified Eagle’s medium with 1% gentamicin^23,24^. 50 µg/ml nanoparticle working solutions were prepared at room temperature, protected from light, and coated with 4.5 µg/ml of a poly-D-lysine solution (Sigma-Aldrich, St. Louis, USA) to ensure DNA binding. Nanoparticles were incubated with 50 ng/ml GCSH (*Tv*1 or Tv*) cDNA for 1 h. Cells were transfected with labeled nanoparticles for 4 h, washed twice with serum-free medium and cultivated up to 24 h in cell culture medium. Following controls were used: 1. Non-transfection control (w/o DNA, w/o nanoparticles); 2. mock control (nanoparticles, w/o cDNA), and 3. GAPDH-control (GAPDH-cDNA, nanoparticles). Transfection efficiency, nanoparticle internalization, and transient expression of GCSH variants were determined by confocal laser-scanning microscopy and flow cytometry.

### Cell viability and cytotoxicity

Cell viability was calculated by colorimetric measurements of metabolic activity with the CellTiter 96®AQueous One Solution Cell Proliferation Assay (Promega Corp., Madison, USA)^25^. Lactate dehydrogenase release was calculated with the Cytotoxicity Detection Kit (LDH) according to the manufacturer´s instructions (Roche Diagnostics GmbH, Mannheim, Germany)^26,27^. At least, eight replicates with corrected background absorbance were conducted.

### Live-cell metabolic monitoring

A real-time live-cell monitoring of three metabolic parameters (impedance, mitochondrial respiration and extracellular acidification) was performed with the Bionas Discovery™ 2500 system (Bionas GmbH, Rostock, Germany) as described previously^28^. Cells were applied to the Bionas Discovery^TM^ SC1000 measurement chips 24 h before measurement in a cell density of 200,000 cells/sensor chip in 450 μl medium. The software CSv1.47 Controlling was used for the raw data acquisition and further analyzed by Bionas15002 Data analyzerV1.07. Controls were normalized and set to 100%.

### DNA binding studies

The northern blotting technique was already described^29^. DIG-labeled GCSH-Tv* and -*Tv*1 RNA-probes were synthesized by *in vitro* transcription with the DIG RNA Labeling Kit (SP6/T7) (Roche Diagnostics, Mannheim, Germany) using T7 RNA polymerase GCSH-Tv* and -*Tv*1 plasmids were linearized by the restriction enzyme MluI (Thermo Fisher Scientific Inc., Waltham, USA). RNA probes (1.5 µg) and 0.5–10 kb RNA Ladder (Life Technologies Europe, Bleiswijk, Netherlands) were denatured for 5 min at 65 °C. After 1% agarose gel electrophoresis with 6.5% formaldehyde solution (Sigma-Aldrich, Munich, Germany) blotting on positively charged nylon membranes (Boehringer Mannheim, Mannheim, Germany) was performed overnight. Thereafter, RNA probes and positive controls (100 ng/µl drop on membrane) were fixed by UV irradiation for 5 min. The prehybridization was carried out in DIG Easy Hyb (Roche Diagnostics GmbH, Mannheim, Germany) at 60 °C for 30 min. 25 ng/ml DIG-labeled RNA probes were denatured at 95 °C and hybridized overnight at 50 °C in DIG Easy Hyb (Roche Diagnostics GmbH, Mannheim, Germany). After 5 min washing in (2×) Saline-sodium-citrate buffer containing 0.1% SDS and 15 min in 0.1x Saline-sodium-citrate buffer containing 0.1% Sodium-dodecyl-sulfate at 55 °C, membranes were blocked (2% FCS, 0.3% Triton, 150 mM NaCl, 0.1 M Tris, pH 7.5). For RNA detection anti-Digoxigenin-AP Fab fragments (Roche Diagnostics GmbH, Mannheim, Germany) were applied in the dilution of 1:5000 and incubated for 1 h at room temperature. The RNA-RNA binding signals were visualized with the ELF®97 Endogenous Phosphatase Detection Kit (Invitrogen, Eugene, USA).

### Quantification of glycine and serine

For the extraction of glycine and serine we followed the protocol of Lisec *et al*.^30^. Briefly, 24 h after transfection 1*10^6^ MCF-7 cells were washed three times with ice-cold 0.9% NaCl and were extracted by lysing cells in 500 µl ice-cold methanol/acetonitrile/H_2_O (50:30:20). Additional 500 μl extraction buffer were used to remove all cell remnants. Samples were shaken at 4 °C for 10 min, then centrifuged for 15 min at 16,000 *g*, and the supernatant was collected and analyzed by LC-MS. The final aqueous supernatant was dried by lyophilization. The dry extracts were dissolved in 200 µl of *A*. *dest*. (ROTISOLV® LC-MS-grade, Roth, Germany) and filtered through 0.2 µm filters (Omnifix®-F, Braun, Germany). The cleared supernatants were analyzed using the high-performance liquid chromatograph mass spectrometer LCMS-8050 system (Shimadzu, Japan) and the incorporated LC-MS/MS method package for primary metabolites (version 2, Shimadzu, Japan)^31^. In brief, 1 µl of each extract was separated on a pentafluorophenylpropyl (PFPP) column (Supelco Discovery HS FS, 3 µm, 150 × 2.1 mm) with a mobile phase containing 0.1% formic acid. The compounds were eluted at 0.25 ml min^−1^ using the following gradient: 1 min 0.1% formic acid, 95% *A*. *dest*., 5% acetonitrile, within 15 min linear gradient to 0.1% formic acid, 5% *A*. *dest*., 95% acetonitrile, 10 min 0.1% formic acid, 5% *A*. *dest*., 95% acetonitrile. Aliquots were continuously injected in the MS/MS part and ionized via electrospray ionization (ESI). The compounds were identified and quantified using the multiple reaction monitoring (MRM) values given in the LC-MS/MS method package and the LabSolutions software package (Shimadzu, Japan). Authentic glycine and serine standard substances (Merck, Germany) at varying concentrations were used for calibration.

### Statistical analysis

All experiments were replicated at least three times with individually passaged cells, and data sets were expressed as means ± standard deviations (SD). Statistical significance was determined by the unpaired student’s *t*-test or one-way ANOVA (***P < 0.001; **P < 0.01; *P < 0.05) using the software Graphpad Prism Version 5 (http://www.graphpad.com/scientific-software/prism/).

## Electronic supplementary material

Supplementary Dataset 1
