# Supplementary material for: GCSH antisense regulation determines breast cancer cells’ viability
Source: Sci Rep. 2018 Oct 18;8:15399. doi: 10.1038/s41598-018-33677-4 (PMC6193953; doi:10.1038/s41598-018-33677-4)

Supplementary Information

**Title: GCSH antisense regulation determines breast cancer cells’ viability.**

Anna Adamus^1,2^, Petra Müller^2^, Bente Nissen^2^, Annika Kasten^3^, Stefan Timm^4^, Hermann Bauwe^4^, Guido Seitz^1^, Nadja Engel^1,2^*

^1^ Department of Pediatric Surgery, University Hospital Marburg, Baldingerstraße, 35033 Marburg, Germany.

^2^ Department of Cell Biology, Rostock University Medical Center, Schillingallee 69, 18057 Rostock, Germany.

^3^ Department of Oral and Maxillofacial Surgery, Facial Plastic Surgery, Rostock University Medical Center, Schillingallee 35, 18057 Rostock, Germany.

^4^ University of Rostock, Plant Physiology Department, Albert-Einstein-Straße 3, 18059 Rostock, Germany.

*Corresponding author:

Nadja Engel, Department of Pediatric Surgery, University Hospital Marburg, Baldingerstraße, 35033 Marburg, Germany.

Tel.: +49 (0) 6421/58-64233 (office), +49 (0) 6421/58-62865 (lab)

Email: (1) nadja.engellutz@staff.uni-marburg.de, (2) nadja.engel-lutz@gmx.de

# Supplementary Figures

**Supplemental Figure** **1.** *Tv** transient overexpression in the breast cancer cell lines BT20, MDA-MB-231 and the non-tumorigenic control cell lines MCF-10A and MCF12A.


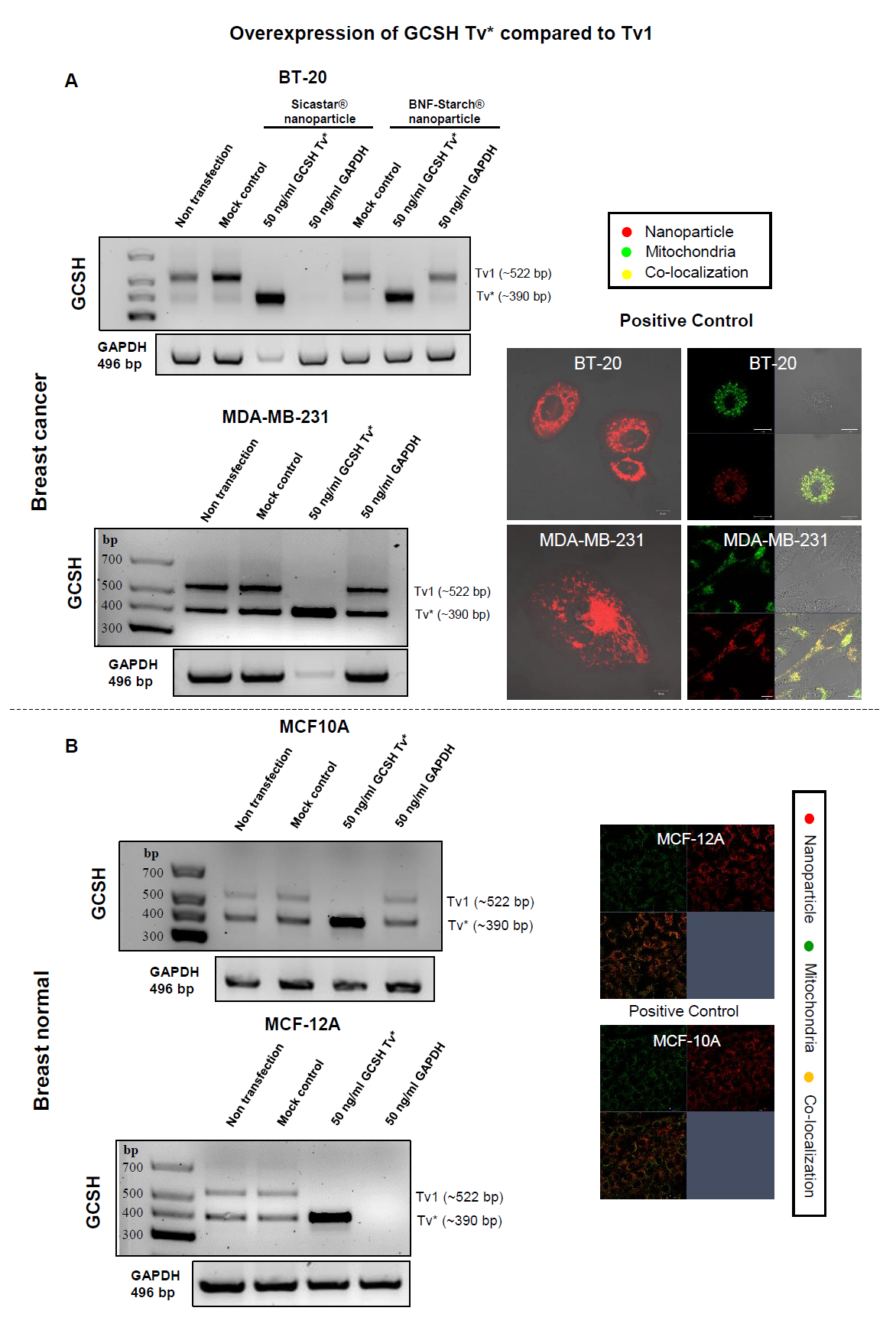


**Supplemental Figure 2.** *Tv** and *Tv*1-GFP plasmids for stable overexpression.


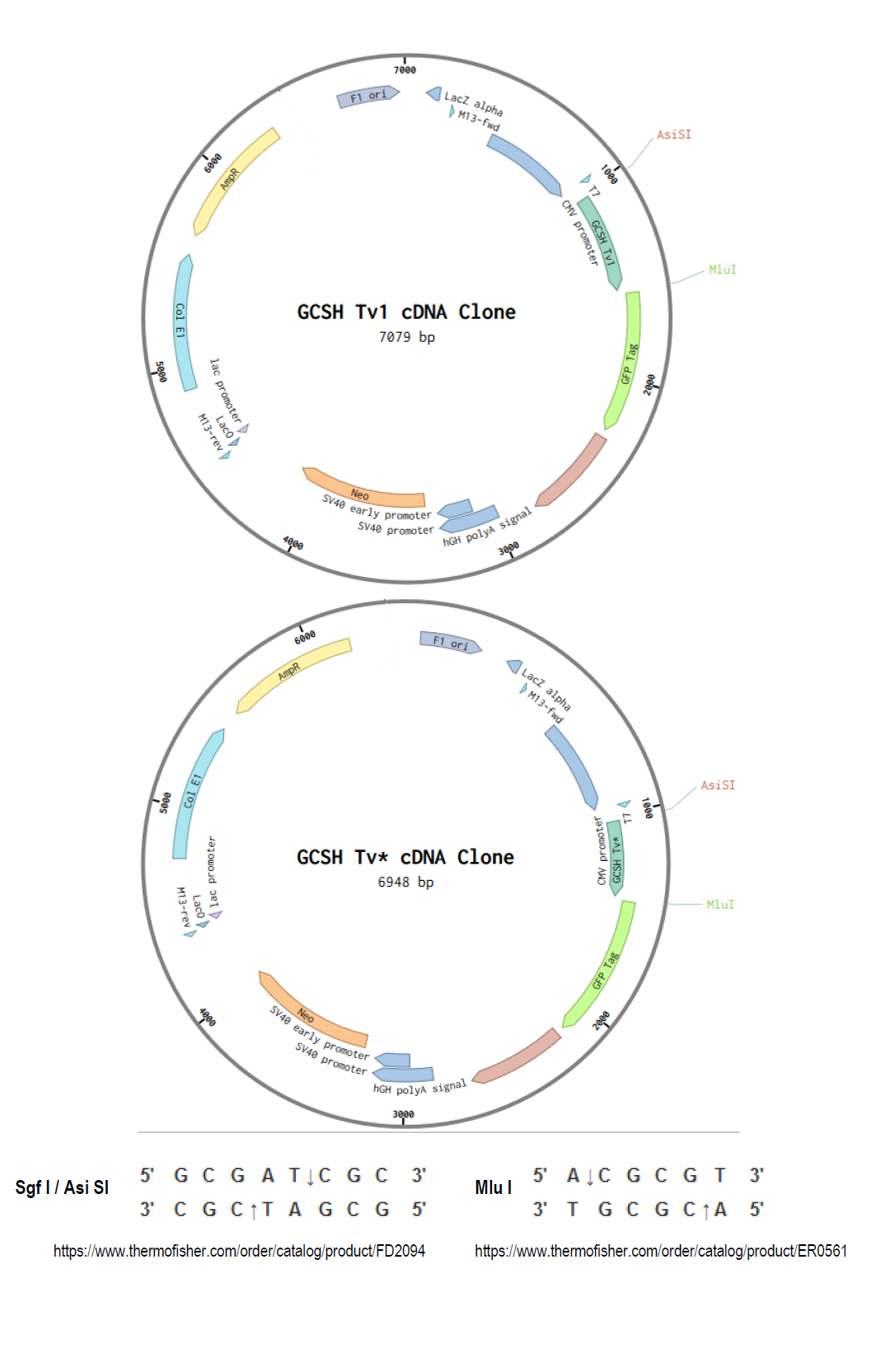


**Supplemental Figure 3.** Long-term survival and relapse-free survival are determined by low GCSH levels. Histograms were generated with the R2 platform (http://r2platform.com / http://r2.amc.nl), a genomics analysis and visualization platform that provides an interface to high throughput data. A: Kaplan-Meyer curve for the correlation of overall survival with GCSH expression. Red: low GCSH expression. Blue: high GCSH expression.


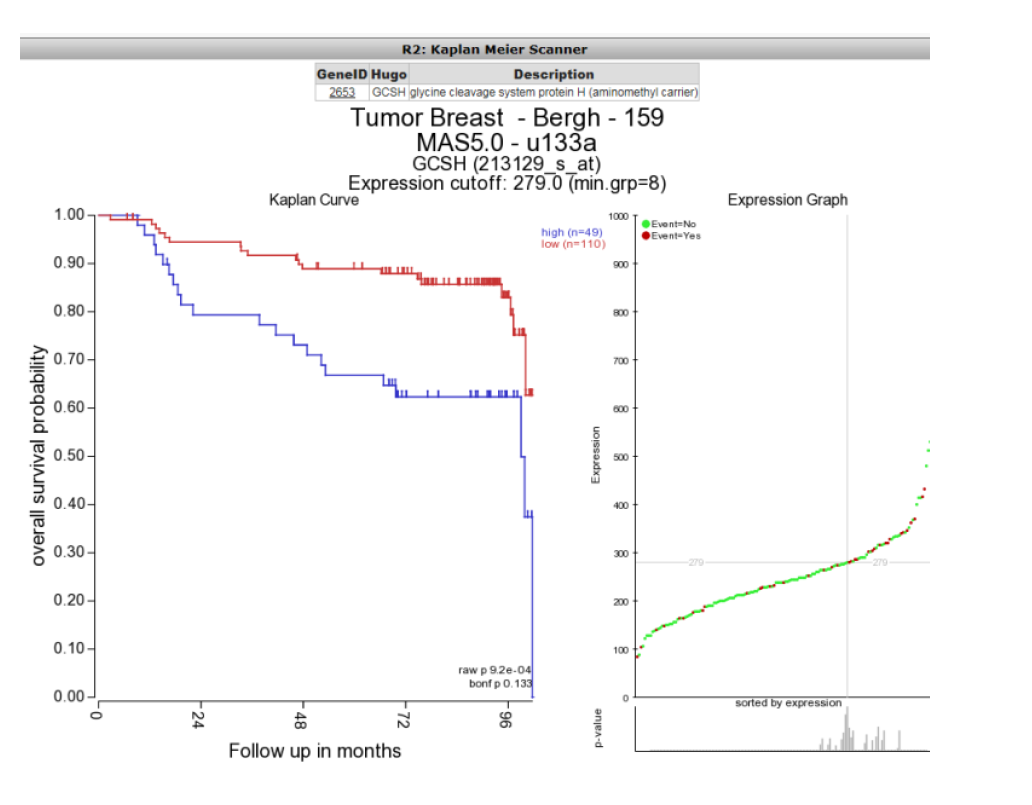


**Supplementary figure 4:** Uncut gels and blots of figure 2.

RT-PCR analysis using GCSH Tv1 specific primers.

PCR: GCSH PCR: GAPDH


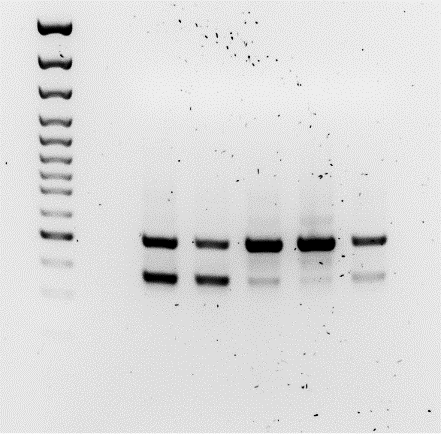

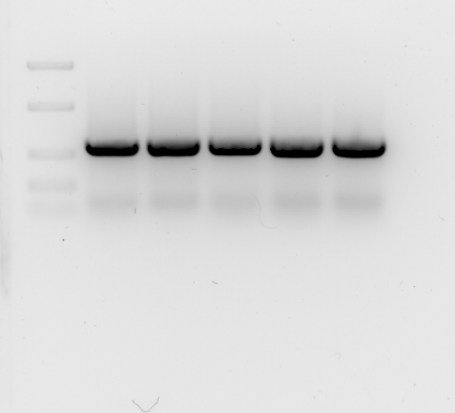


GCSH, PCNA, ß-Actin and AMT protein content in all 5 cell lines was visualized by western blotting.

Western Blot: GCSH Western Blot: PCNA


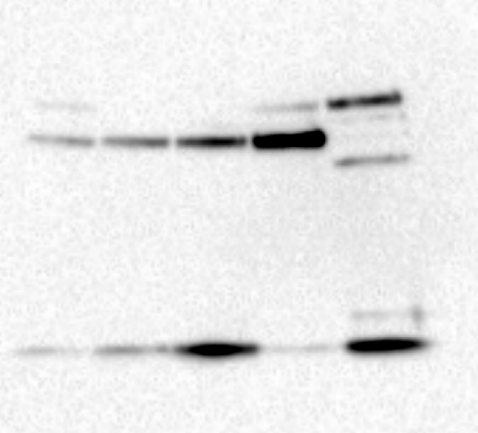

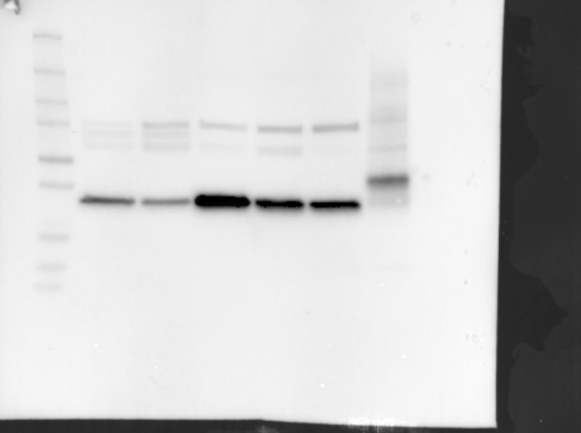


Western Blot: ß-Actin Western Blot: AMT


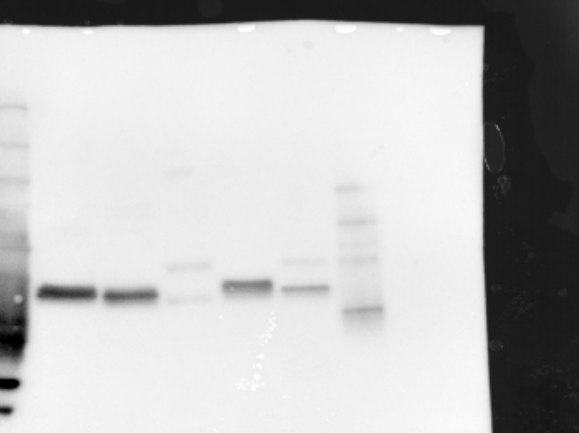

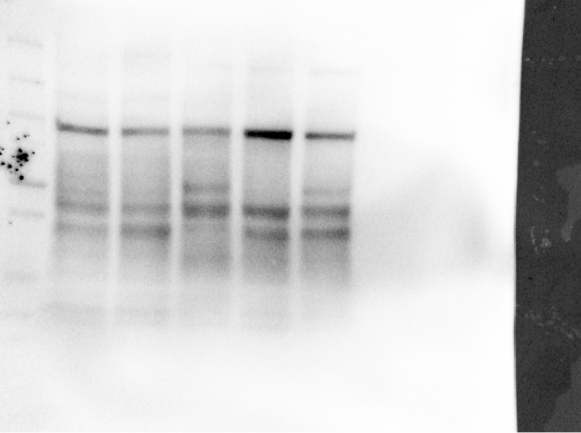


**Supplementary figure 5.** Uncut gels and blots of figure 3.

Northern blotting demonstrated a positive antisense binding of the Tv* to isolated RNA of the cancer cell line MCF-7.

**
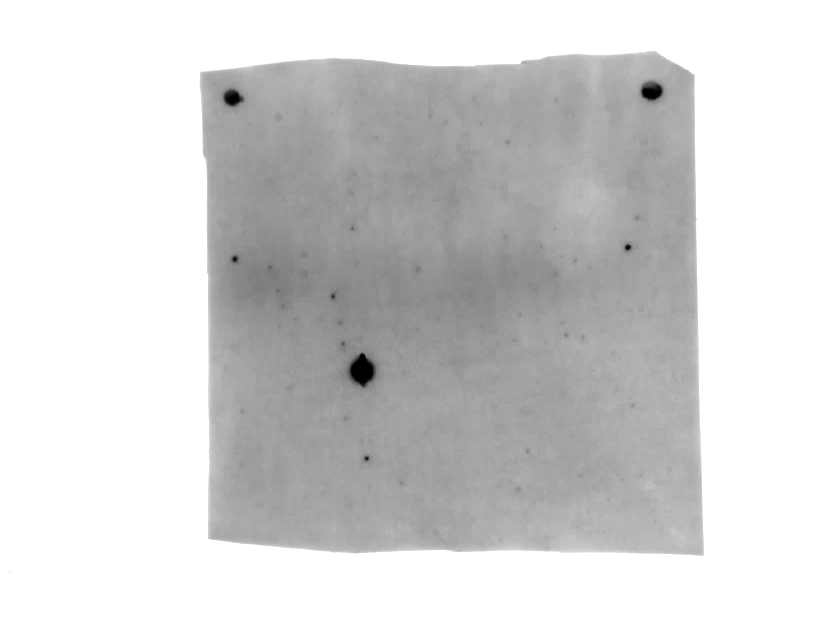
**

**Supplementary figure 6.** Uncut gels of figure 4.

1. Evidence of the stable binding between Tv* and nanoparticles. All of the 50 ng Tv*-DNA was completely bound to the nanoparticles which cannot pass the agarose gel.


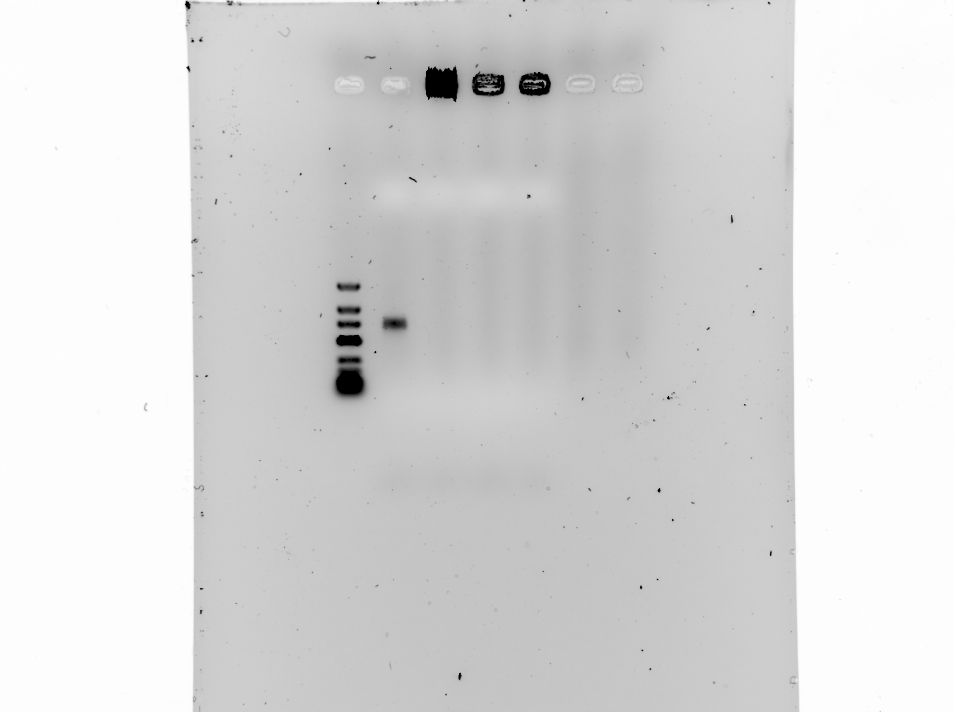


1. RT-PCR of GCSH transcripts Tv1 and Tv* of non-transfected (n.t.), mock-transfected (C), Tv*-transfected or GAPDH-transfected MCF-7 cells. Results were similar with silica and magnetic nanoparticles: Tv*-overexpression decreased Tv1 expression, and Tv* was found as the most prominent band. In general, control transfection (nanoparticles, w/o DNA) did not alter the GCSH transcript profile.


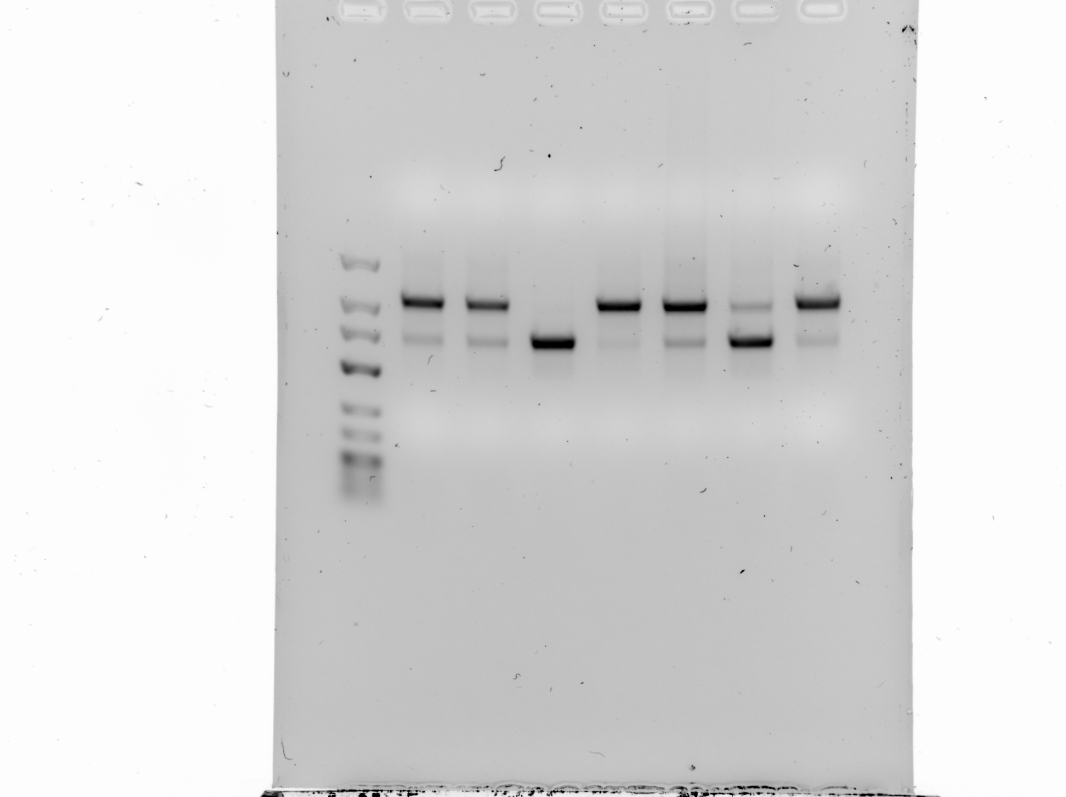


**Supplementary figure 7.** Uncut gels of figure 5.

**PCR: GCSH and GAPDH**

RT-PCR of GCSH transcripts Tv1 and Tv* of non- (n.t.), control- (C), Tv*- and Tv1-transfected MCF-7 cells. Tv*-overexpression revealed comparable results to nanoparticle mediated transfection: low Tv1 and high Tv* signals. Surprisingly, Tv1-overexpression did not increase the Tv1 content. Furthermore, no Tv* amplificat could be detected but instead a third ~ 300 bp transcript variant (Tv?) appeared.

**
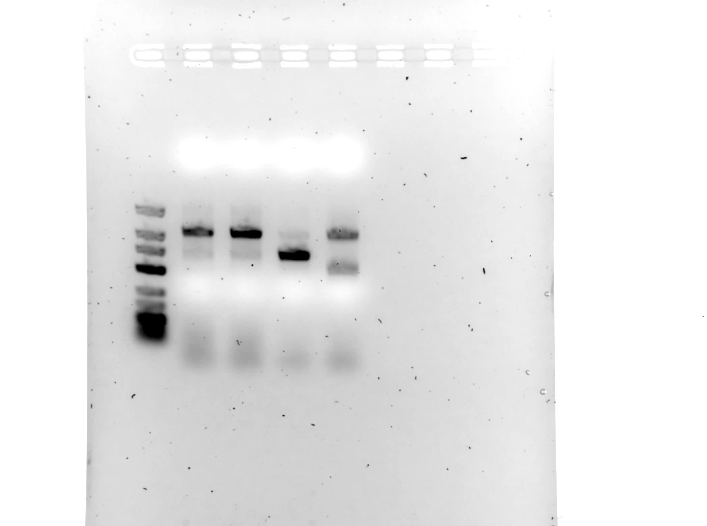

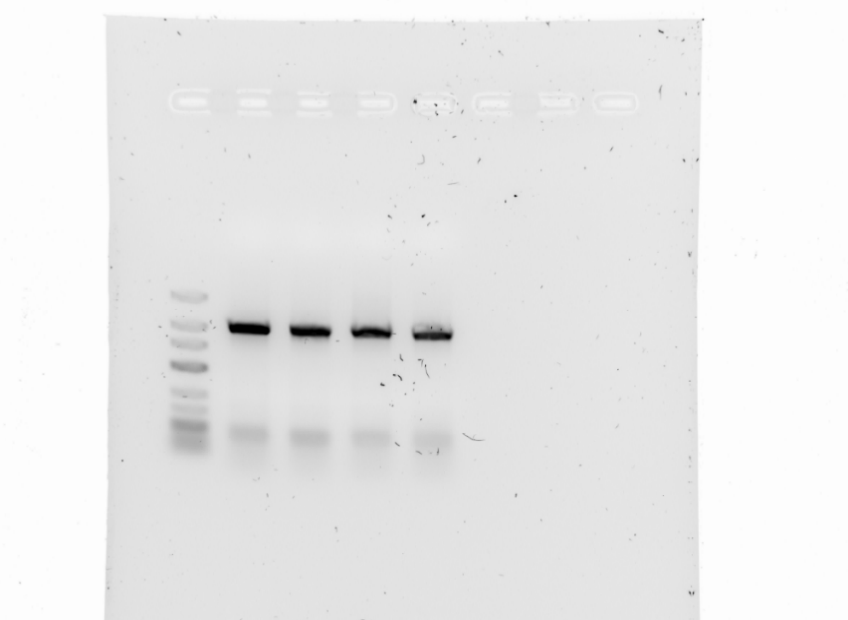
**

**Supplementary figure 8.** Uncut western blots of figure 5.

Immuno blots of GCSH, AMT and ß-actin proteins in non-treated (n.t.), mock-treated (C), three individually transfected Tv1- and Tv*-overexpressor MCF-7 cell populations.


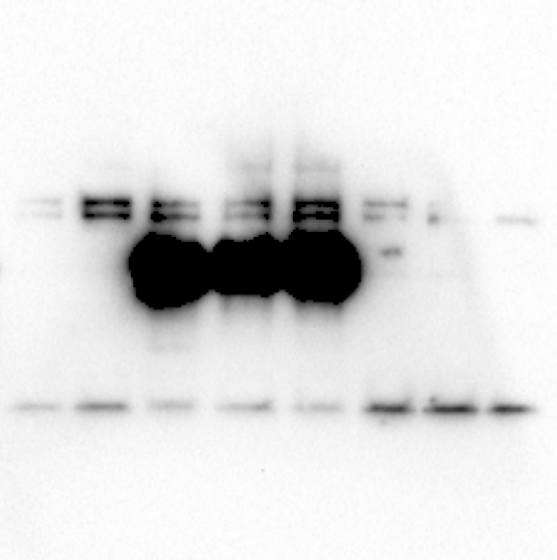
 **
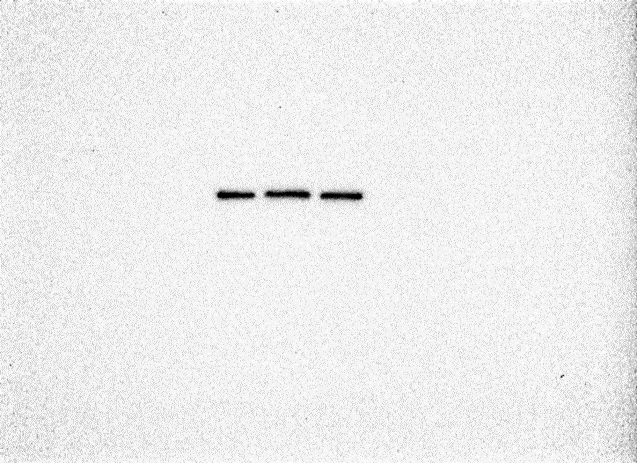
**

**
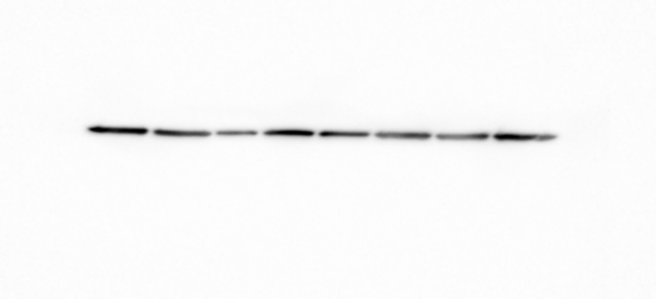
**

Western Blot: cSHMT Western blot: mSHMT


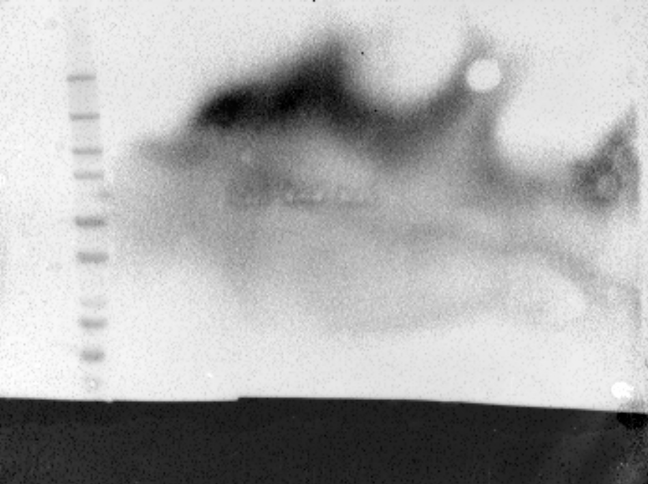

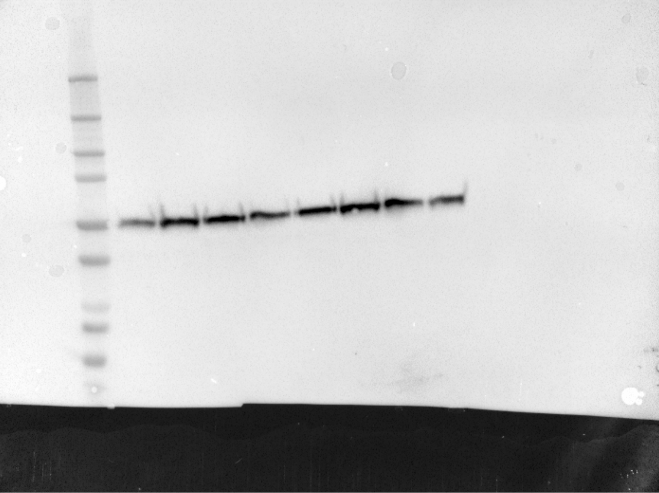


Western blot: GNMT


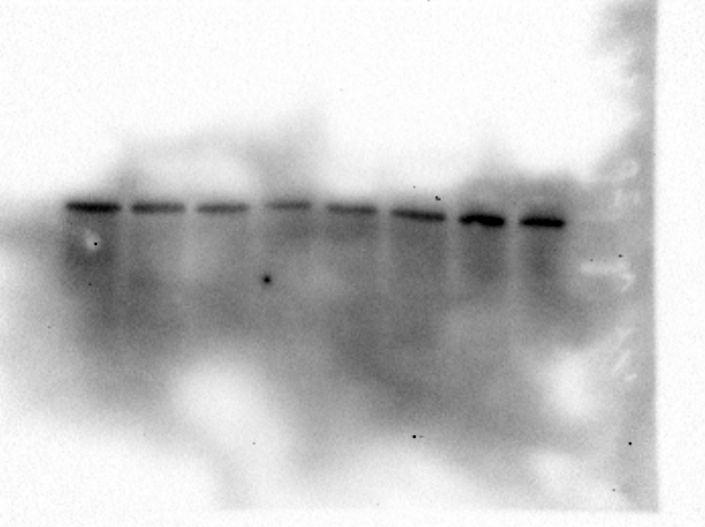

Supplement: Supplementary file 1 — Supplementary Dataset 1 [file 41598_2018_33677_MOESM1_ESM.docx]
